# Comparative analysis of volumetric changes between resection volume of oral tongue cancer and post operative volume of radial forearm flaps

**DOI:** 10.1007/s00784-024-05885-y

**Published:** 2024-08-25

**Authors:** Matthias Zirk, Mina Niknazemi, Maximilian Riekert, Matthias Kreppel, Christian Linz, Max-Philipp Lentzen

**Affiliations:** 1 Department for Oral and Craniomaxillofacial and Plastic Surgery, University of Cologne, Cologne, Germany; 2 Medical Faculty, University of Cologne, Cologne, Germany; 3 Oral and Craniomaxillofacial and Plastic Surgery, MKG Köln West, Bunzlauerstr. 1, 50858 Cologne, Germany

**Keywords:** Oral tongue cancer, Radial forearm free flap, Squamous cell carcinoma, Computed tomography, Magnetic resonance imaging, Depth of invasion, Ulnar forearm free flap, Volumetric measurements, ITK-SNAP

## Abstract

**Objectives:**

This study investigates the relationship between the total volume of oral tongue cancer pre-operatively and the RFFF volume post-operatively.

**Materials and methods:**

A total of 52 DICOM imaging datasets (CT or MRI) of 26 patients were included in this study. The volume of the desired structure was quantified using semi-automatic segmentation using the software ITK-SNAP. All extracted measurements were validated by two further clinicians at separate instances.

**Results:**

The variation of MeanVolTu can be predicted by MeanVolFlap moderately reliable with 59.1% confidence (R-Qua: 0.591). ANOVA Testing to represent how well the regression line fits the data, resulted in the overall regression model being statistically significant in predicting the MeanVolTu (*p* < 0.001). The flap volume may be predicted using the following algorithm: MeanVolFlap0 = 3241,633 + 1, 322 * MeanVolTu.

**Conclusion:**

The results of this study show positive correlation between tumor volume and flap volume, highlighting the significance of efficient flap planning with increasing tumor volume. A larger extraction volume of the radial forearm free flap from the donor site compromises the forearm more, thus increasing the probability of post-operative complications.

**Clinical relevance:**

Radial forearm free flap design in accordance with its corresponding 3D tumor volume.

## Introduction

The tongue muscle fulfils crucial roles within the human body, which can be divided into motor and sensory functions. The motor functions include grasping, grinding, and passing of the food bolus during mastication, as well as speech and language. Specialized tastebuds located within the surface mucosa of the tongue, allow the sensory function of tasting. Furthermore, the tonsilla linguae act as a barrier to pathogens entering through the oral cavity [1].

Over the past decades confirmed cases of oral cancer have continuously increased, currently accounting for a worldwide yearly incidence of 200.000–350.000 [2, 3]. With a total share of 30%, tongue cancer presents itself as the most common malignant cancer of the oral cavity [4, 5].

Once TNM Staging has been conducted by the tumor board, a treatment plan is designed which may consist of a given combination of surgery, radiation therapy, and chemotherapy. The choice of treatment will depend on the TNM stage and location of the cancer, as well as the overall health of the patient [6].

The main aim of the treatment is to achieve local control of the tumor with as little functional or esthetic impairment as possible. The primary goal in free flap reconstruction of the tongue is to preserve the mobility of the organ and to maintain its muscle function. By replacing resected parts of the tongue using innervated vital muscle flaps (infrahyoid pedicled muscle flap), the function is maximized [7].

Surgical resection of the tumor with negative margins is an essential strategy for the curative treatment of SCC of the tongue. A resection with negative margins consists of the complete excision of the primary tumor with no residual cancer cells left behind [8–10].

If the resulting tongue defect that occurs after surgery cannot be healed secondarily, a reconstruction can be performed using regional or free flaps. The chosen reconstruction method and the origin of the flap depend on various factors including the size of the defect, the exact location, which tissues are being replaced by the flap and the policy of the respective institution [11]. The RFFF is the most used microvascular flap when the restoration of tongue mobility is the primary goal of the reconstruction [12, 13]. Whilst numerous donor site characteristics must be considered, such as possible function loss in the arm including pinching and gripping strength or subsequent skin graft coverage, the RFFF still outclasses other free flap options in terms of functionality [13, 14].

Due to the well-known phenomenon of post-operative free flap shrinkage, a slightly larger volume is extracted from the donor site to compensate this decrease in volume [16–18]. There may be multiple factors influencing the volume of a free flap, such as adjuvant radiotherapy [18–20], muscle atrophy (for muscular flaps) due to denervation [21], transitional ischemic effects [22, 23], as well as edema, hematoma and inflammation after surgery [24, 25]. Further to this the quality of recipient vessels as well as the choice of vessels may further influence the amount of flap shrinkage [26].

At present there is no scientific literature that investigates the correlation of pre-operative tumor volume and post-operative flap volume of SCC of the tongue. A suitable surgical plan in the treatment of oral tongue cancer should include an accurate interdisciplinary staging, a well-structured strategy to cover defects after tumor resection successfully as well as proper consideration of the donor site. The efficiency and success of a treatment program may vastly benefit from accurate planning that can predict extraction volumes, thus decreasing donor site complications and improving the patient care provided. The aim of this retrospective pilot study was to conduct a comparative analysis between resected oral tongue cancer volume and post-operative volume of the radial forearm free flap to aid surgical flap planning and improve patient care.

## Materials and methods

Within this retrospective pilot study, a total of 52 DICOM imaging datasets of 26 patients (7 females, 19 males) of the University clinic of Cologne were identified, which met the criteria to be included in this study. All patients, who had been issued a diagnosis of oral tongue cancer (ICD10: C01, C02, C04, C06, C14) from 01.01.2009–01.06.2021, were considered for this study. Further descriptions of ICD10 classifications which described cancerous lesions in areas other than the corpus of the tongue (for instance cancer of the floor of the mouth), were used for exclusion of the data. The analysis of patient records and surgical reports of the remaining 105 patients lead to a total sample of 70 patients, who received an RFFF after tumor resection. Using the medical imaging Software of the University clinic of Cologne (ImpaxEE) the remaining datasets were filtered according to pre- as well as post-surgical (MRI or CT) clinical diagnostics performed within the radiology department of the University clinic of Cologne. 42 patients in total had both their pre- and post-surgical images available on the system, of which 16 further datasets had to be excluded from this study due to imaging artefacts, post operative complications (fistulas, hematoma) or flap failure leading to premature removal of the flap, thus providing a data pool of 26 datasets in total. The T-classification of the included candidates is constituted of T1 (*n* = 10) and T2 (*n* = 16) cases.

Within ITK-SNAP [27] the sagittal, coronal, and axial slices were merged, and a three-dimensional image obtained. MRI and CT images were acquired with voxels of equal size in each plane (isotropic spatial resolution). Using semi-automatic segmentation of the pre-surgical images, the tumor was then identified and volumetric calculation in cubic millimeters carried out by ITK-SNAP. To decrease error within the measurements, each semi-automatic segmentation was carried out 3 times on the same imaging set in a standardized manner by repeating each measurement at three independent instances. Leaving sufficient time between the measurements aids the evaluation of the same imaging data set with a fresh mind rather than their fabrication due to remembrance of repeating structures.

The mean values of the datasets were then used for further statistical analysis. This method was adapted for each patient using semi-automatic segmentation of post-surgical images, thus identifying the volume of the RFFF. All imaging dataset measurements were validated by two further maxillo-facial surgeons on separate instances (minimum of 48 h between each measurement).

The datasets were summarized initially using Excel spreadsheets and analyzed statistically using the Program IBM SPSS Statistics for MAC (IBM SPSS Statistics for MAC, version 28.0.1.1.(14); IBM, Armonk, NY). Categorial variables were investigated in terms of frequency and mode. The Mann-Whitney-U-Test for independent samples was used to determine distribution patterns within age and gender. Further analysis included the calculation of mean, median, standard deviation and percentiles for numerical variables including tumor volume and flap volume. The data was analyzed for normal or abnormal distribution patterns, whereby the tests showed the presence of one outlier value for tumor volume. After considering the plausibility of the increased tumor volume value and considering it’s dependency on the extent of disease within the patient, the outlier was included in the data. This led to the data being abnormally distributed and conducting the Spearmen correlation test for rejecting or accepting the null hypothesis. The Friedman test was used to ensure accuracy of the repeated measurements for Tumor Volume and Flap Volume. Regressions tests were carried out to investigate correlations between abnormally distributed variable values. Statistical variations were considered significant if their occurrence could be reasoned by a minimum of 95% due to a factor other than chance, thus resulting in *p* < 0,05.

Relevant pre- and post-surgical imaging series were selected, and their corresponding DICOM data sets anonymously exported from ImpaxEE and imported into the open-source software ITK-SNAP (Penn Image Computing and Science Laboratory). All personalized data was removed from the imaging datasets, providing fully anonymized images for volumetric quantification.

The study was approved by the Ethics Committee of the University Hospital Cologne (No 23-1444-retro). All procedures performed in the study were in accordance with the 1964 Helsinki declaration and its later amendments.

## Results

**Fig. 1:**
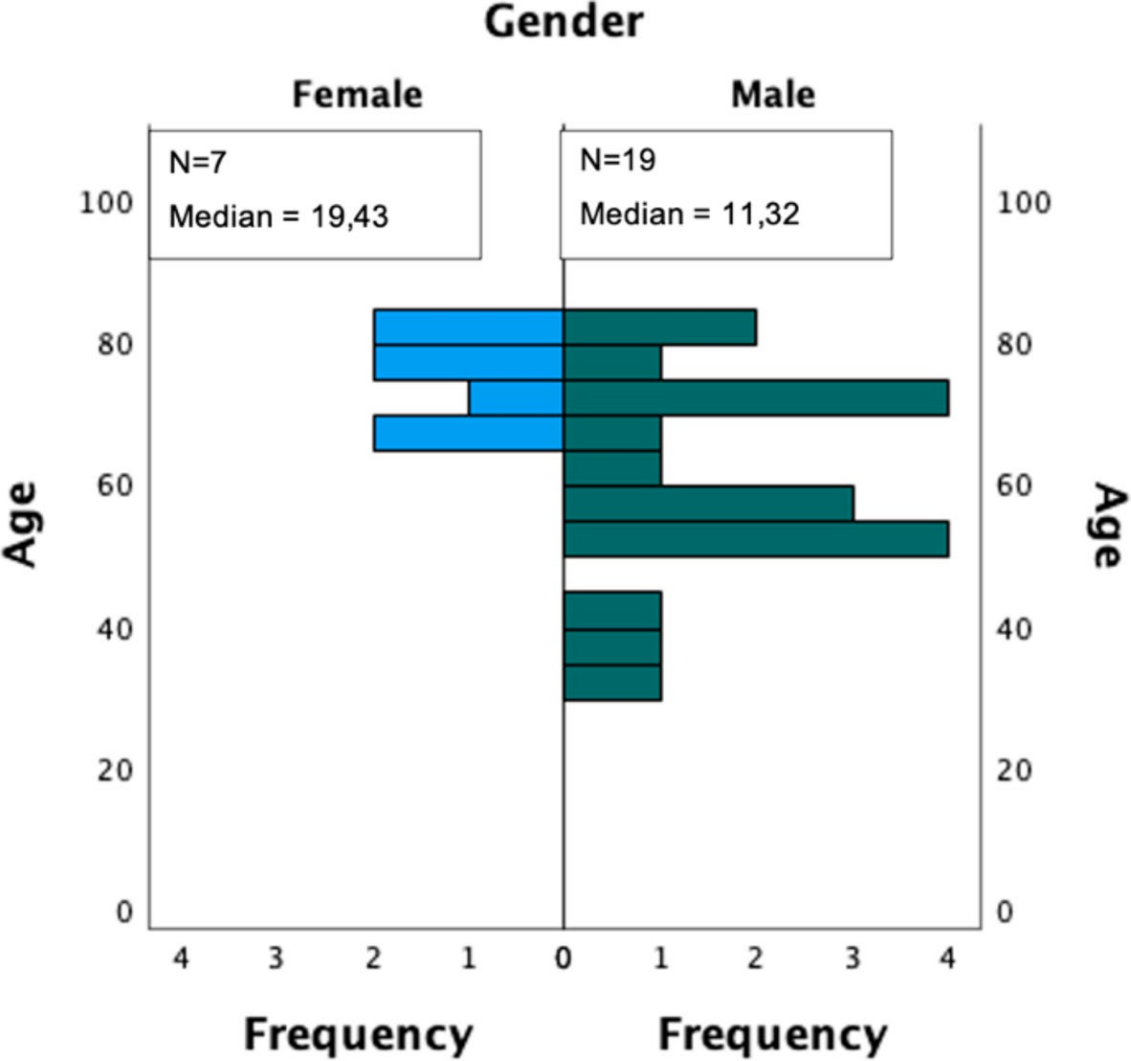
Age distribution of male and female subjects

The subjects age ranged between 32 and 84 years. The resulting mean age was 64 (± 14,9 SD). 19 subjects (73,1%) were male, whilst 7 subjects (26,9%) were female, this displaying a strong trend towards male subjects. The distribution of age according to gender is illustrated within a side-by-side boxplot in Fig. 1.

To elaborate whether the age distribution of male and female showed any statistically significant differences, the Mann-Whitney-U Test for independent samples was used. Considering the null hypothesis as age distribution being the same for both female and male gender, the results of this analysis reject the null hypothesis (*p* = 0,015). Thus, the study presented a male gender dominance.

**Fig. 2:**
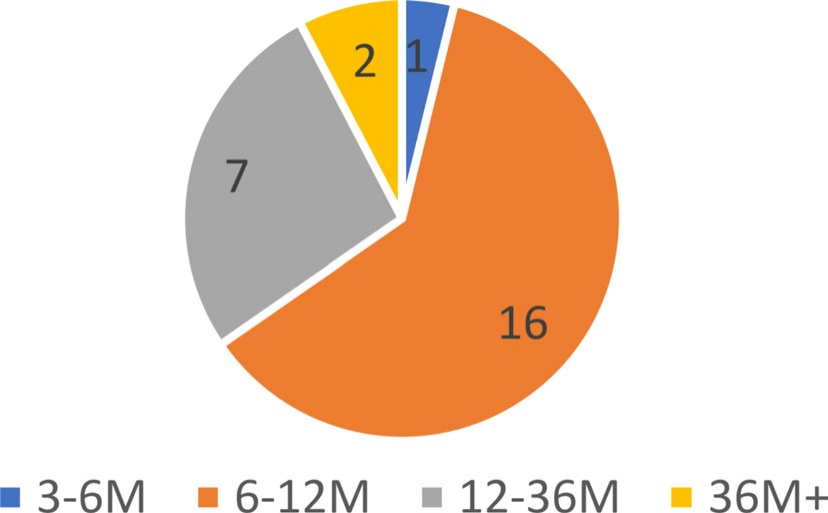
Time between pre-operative scan of tumor and post-operative scan of flap in months (M)

Figure 2 displays an overview of the time between the scans of the pre-operative tumor and the post-operative flap. A total of 16 subjects received their postoperative scan within 6–12 months after their preoperative scan being taken. This accounts for over 60% of the total patients within this study.

The pre-operative imaging data on ImpaxEE for each patient was analyzed in different planes. The sections which provided most visible margins of the tumor were exported as DICOM images and uploaded onto ITK-SNAP. Using semi-automatic segmentation, the original tumor Volume prior to resection was calculated by the program. The measurements were repeated three times with at least 24 h between each measurement of the same dataset. The validation of these results was conducted by two further clinicians at independent variables.

**Fig. 3:**
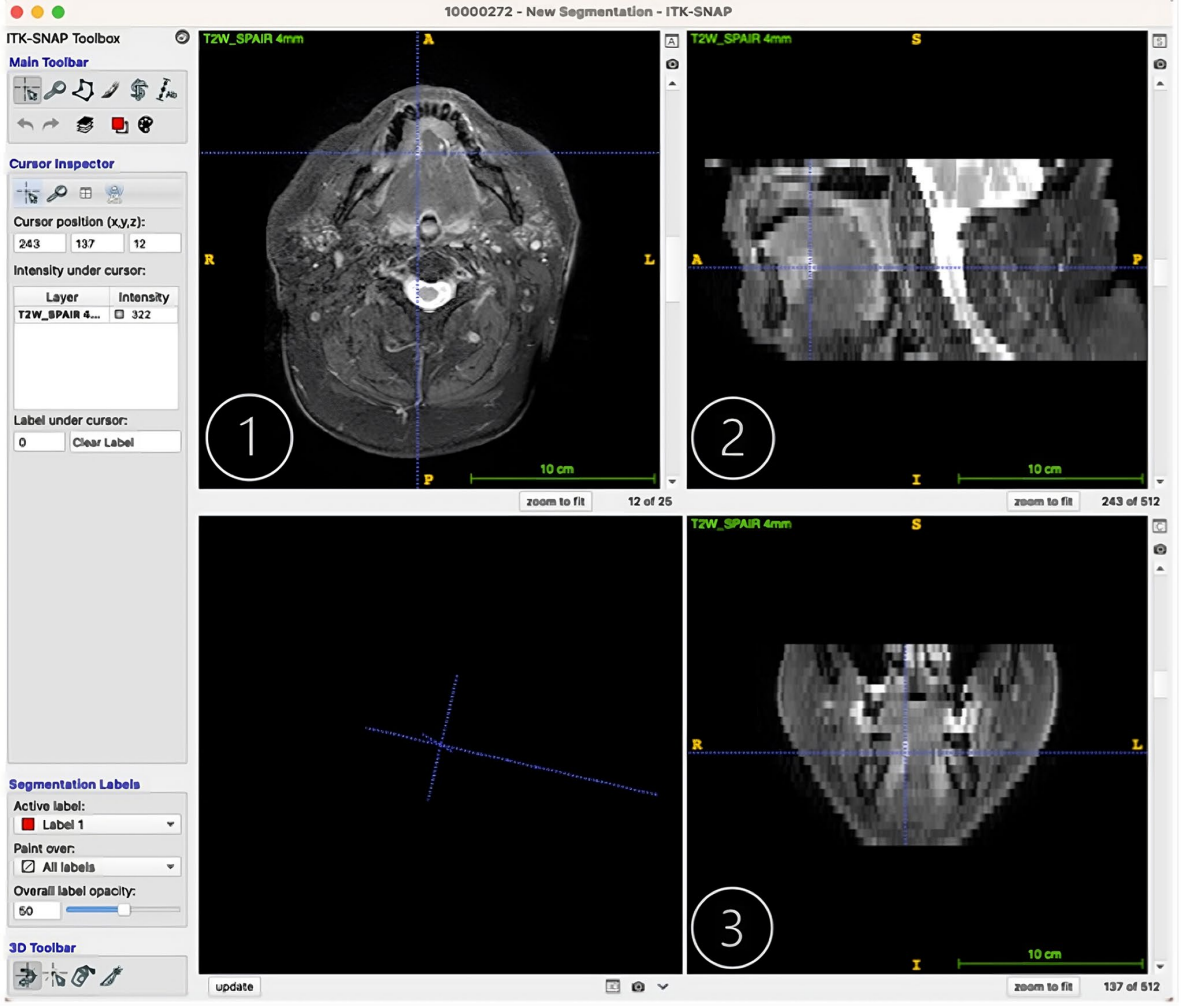
Editorial view of a pre-operative imaging dataset on ITK-SNAP. Each window represents another plane: **1**- transversal, **2**- sagittal, **3**- frontal [28]

The editorial view of a pre-operative imaging dataset in differing planes: 1- transversal, 2- sagittal, 3- frontal on ITK-SNAP, is illustrated below in Fig. 3 [28].

After marking the tumor mass in all planes using semi-automatic segmentation, the total volume of the cancer prior to the operation was calculated using the “Volumes and Statistics” toolbar within the program. Using the “active contouring” tool, the tumor mass was extracted from the surrounding structures and displayed in isolation. Within this viewing modus the mass can be observed from different angles by moving the image around inside the application. Once all tumor volumes prior to the operation of all 26 patients were determined, the values for minimum, maximum, mean Volume, Variance and SD (Standard deviation) were calculated as displayed below in Table 1.

**Table 1:** Summary of results for tumor volumes (in mm³) prior to operation

	VolTumor1	Voltumor2	VolTumor3	MeanVolTu
Mean	4347,96	4551,42	4606,62	4502,0008
Median	3587,50	3452,50	3875,00	3716,8350
Standard Deviation	2572,668	2932,010	2829,595	2743,78291
Minimum	1496	1208	1742	1527,00
Maximum	13,250	14,220	15,050	14173,33

The standard deviation (SD) and Variance was also determined. As a result of this, the mean tumor volume was found to create a mass of 4502,00 (± 2743,78 SD) mm³.

In an analogue manner, the volume for flap was identified. Once all RFFF volumes after the operation of all 26 patients were determined, the mean Volume was calculated.

**Table 2:** Summary of results for RFFF volumes (in mm³) after the operation

	VolFlap1	VolFlap2	VolFlap3	MeanVolFlap
Mean	9200,31	9162,42	9214,46	9192,3981
Median	8701,00	8700,50	8953,00	8735,1700
Standard Deviation	4823,667	4823,945	4635,198	4716,16564
Minimum	3086	4359	3660	3701,67
Maximum	23,110	24,720	24,110	23980,00

The above shown Table 2 summarizes the minimum, maximum and mean flap volumes. The standard deviation (SD) and Variance was also determined. As a result of this, the mean flap volume was found to create a mass of 9192,40 (± 4716,17 SD) mm³.

The Friedman test was used to elaborate whether using a mean value of VolTumor1, VolTumor2 and VolTumor3 can be considered as accurate. There are no significant differences in mean volumes of VolTumor1, VolTumor2 and VolTumor3 (*p* = 0,341). The same method was used to elaborate whether using a mean value of VolFlap1, VolFlap2 and VolFlap3 can be considered as accurate. There are no significant differences in mean volumes of VolFlap1, VolFlap2 and VolFlap3 (*p* = 0,962). In summary, the mean volume for tumor and the mean volume for flap may be used for correlation and regression testing. Via SPSS the correlation of 0,769 (*p* < 0.001) shows a highly positive correlation between MeanVolTu and MeanVolFlap. Thus, MeanVolTu increases when MeanVolFlap increases. To examine whether flap volume can be predicted by tumor volume, a regression test for abnormally distributed data was carried out. Considering the mean volumes for both variables, regression testing was conducted via SPSS for MeanVolFlap as the dependent variable and MeanVolTu as the independent variable. The variation of MeanVolTu can be predicted by MeanVolFlap moderately reliable with 59.1% confidence (R-Qua: 0.591). ANOVA Testing to represent how well the regression line fits the data, resulted in the overall regression model being statistically significant in predicting the MeanVolTu (*p* < 0.001). The coefficient values within the regression tests allow the prediction of a regression model, predicting the flap volume using the tumor volume. Thus, the flap volume (MeanFlapVol0) may be predicted using the following algorithm:

$${\bf{MeanVolFlap0}}{\rm{ }} = {\rm{ }}{\bf{3241}},{\bf{633}}\, + \,{\bf{1}},{\rm{ }}{\bf{322}}\,*\,{\bf{MeanVolTu}}$$

**Fig. 4:**
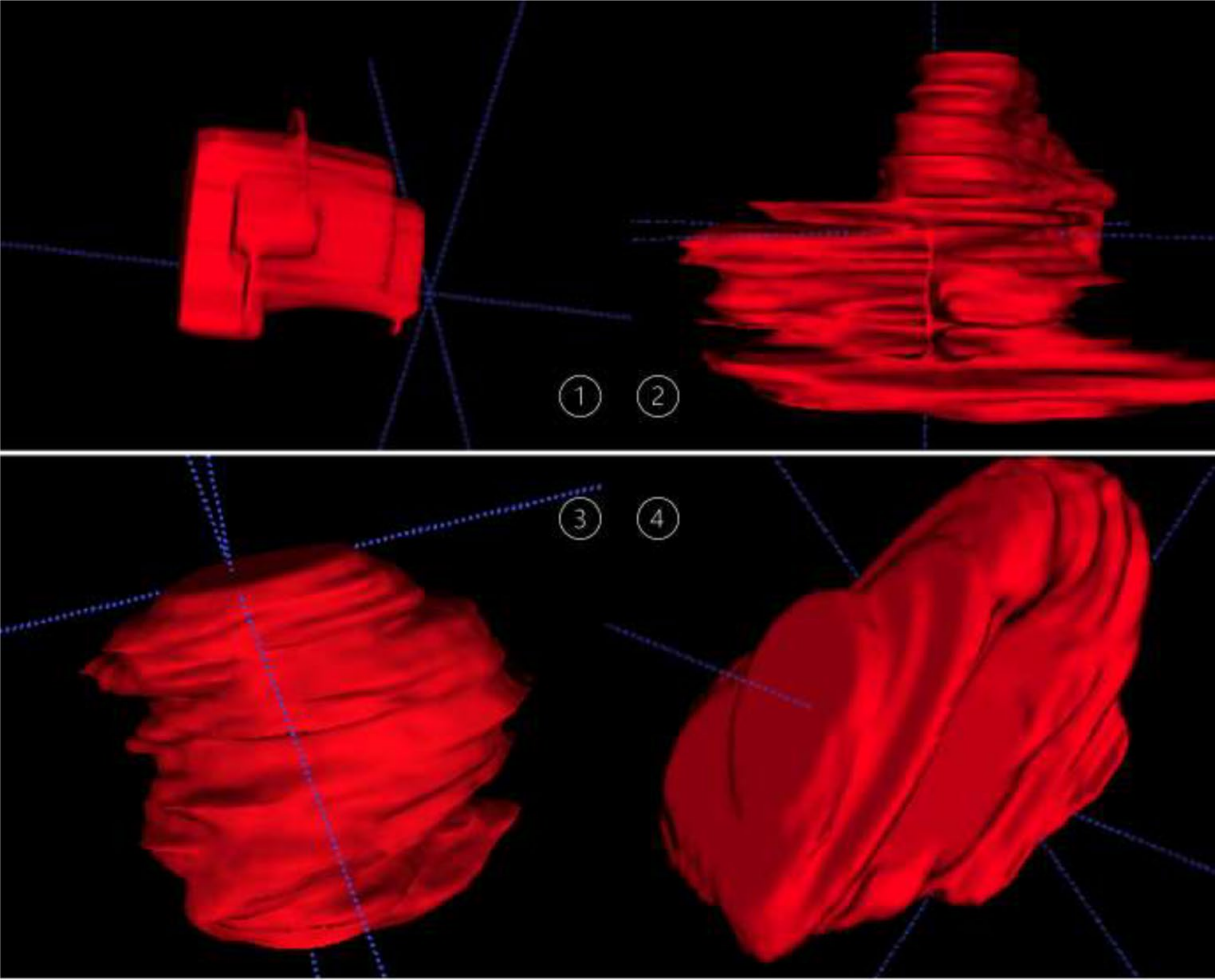
Snapshots of segmentation images: **1**- RFFF, **2** – tumor, **3** & **4** – different angles of the same RFFF [28]

The below shown Fig. 4 summarizes Snapshots of segmentation images for a 3-Dimensional illustration of 1 - an RFFF, 2 – a tumor, 3 & 4 – different angles of the same RFFF [28].

## Discussion

Within this retrospective pilot study, a total of 26 imaging datasets of preoperative tumor volume and the corresponding postoperative flap volume were analyzed. The CT or MRI datasets were measured out at three separate instances, increasing the reliability of these results. The volumetric measurements were validated by two further maxillo-facial surgeons independently. Using semi-automatic segmentation on ITK-SNAP, the required structures were identified, and their volumes calculated. Semi-automatic segmentation has proven to be a reproducible and accurate quantification method for tumor volume [27]. Whilst tumor volume quantification using semi-automatic segmentation on ITK-SNAP has not yet been utilized for tumor and flap quantification in oral tongue cancer, the method has been successfully conducted on hard and soft tissue by various authors including the calculation of volumes of retrobulbar hematoma [29], medication-related osteonecrosis of the jaw (MRONJ) lesions [30] and 4D flow MRI angiographic data for the assessment of carotid bulb hemodynamics [31]. Whilst border measurements of structures on imaging data within ITK-SNAP may be subject to slight variances depending on the individual evaluating them, the use of semi-automatic segmentation increases the reproducibility of this method [28]. The semi-automatic nature of this technique allows for computerized errors due to for instance artefacts to be corrected and combine the best of manual and automatic segmentation. In a clinical study by Gau et al., the authors found that semi-automatic segmentation provided the highest accuracy in resected brain areas with a moderate amount of human input, compared to manual and fully automatic segmentation on ITK-SNAP [32].

To allow more powerful and accurate conclusions the sample size was increased by repeating each measurement at three independent instances. Leaving sufficient time between the measurements aids the evaluation of the same imaging data set with an indifferent view rather than fabrication due to remembrance of repeating structures. Further studies carried out within this field of research would greatly benefit from the involvement of a specialized radiologist for the determination of initial tumor volume. To improve comparability, a standard layer should be selected to avoid discrepancies in findings due to the use of different axes in measurements.

The individual values for volume of the tumor are dependent on various factors, such as the progression of the tumor in the measurable terms of the T-stage of the TNM-Classification, age, gender and many more [11, 33, 34]. Similarly, the volume of each flap is dependent on its initial tumor volume with a positive correlation displayed in the results of this study. For this reasoning the individual cases are compared by analyzing their correlation rather than the individual numeric values. Factors influencing this correlation may include the progression rate of the individual disease, the time passing between pre-operative imaging and operation for tumor volume and the time between the operation and post-operative imaging for flap volume [34]. Also worth mentioning are the variances between the time passing from the operation and post-operative imaging for individual cases. Due to differing patient circumstances and scheduling of the appointment in the appropriate department depending on their adherence, this waiting time may be prolonged. A prolonged waiting time between the operation and the post-operative aftercare imaging, may lead to a decreased flap volume due to flap shrinking [25, 35].

A further aspect of significant clinical importance entails the concept of shared decision making, which originally pioneered within the field of breast reconstructive surgery. Previous studies have shown that the utilization of decision aids lead to an enhancement in decision quality, a decrease in decisional conflict and increased patient satisfaction [36, 37].

Personal aesthetic preferences of patients are greatly influenced by their viewpoint on donor site scars and may impact their overall experience with reconstructive surgery [38].

The tumor volume from each pathological report for the resected tumor could not be used as a comparative variable, due to the R0 resection of the tumor. This means that the surgical margins must be in microscopically tumor-free tissue. As a result of this, the resected mass is greater in its volume than the mere tumor mass so that the pathological volume differentiates itself from the calculated tumor volume from the imaging data. Incorporating these surgical safety margins, thus account for uncertainties in predicting final resection size. On the contrary, irregular flap contractions cause inconsistencies in the opposite direction with irregularly decreasing flap volumes compared to pathologically resected mass volumes. Individual variations in the healing processes as well as Oedema may cause a natural shrinking process of flaps used in reconstructive surgery. For the outlined reasoning the volumes from the pathological reports were excluded from this study.

We have provided an overview of the time between preoperative imaging of the tumor and postoperative imaging of the flap in months, to increase clinical significance. Since the follow-up time influences the volume loss of the flap, an improvement for future study designs suggests a constant imaging protocol. The retrospective nature of this feasibility study focused on the plausibility of this topic. In addition to this, any adjuvant radiotherapy in advanced oral tongue cancer leads to further volume reduction of the flap itself but also surrounding tissues [18–20]. Kim et al. investigated the effect of several variables, including post-operative radiation, on flap volume. The results of this study showed there is no statistically significant change in flap volume between 3 months and 3 years. Since all post operative imaging was taken within 3 months and 3 years for the patients within this study, the radiation data was not considered further [39].

With excellent results within the recipient site of the RFFF over the past decades [40], the importance of good closure of the primary defect of the donor site in terms of functionality and esthetics increases [41]. Most commonly, the primary defect may be covered using a split-thickness skin graft (STSG). Defect coverage with skin grafts in major operations may show a failure rate of up to 28% and are associated with complications such as extended healing times, exposed flexor tendons, or further surgery on the donor site [42, 43]. Doubtlessly, more precise flap size predictions can aid minimize the volume of donor site flap extraction and decrease donor site morbidity. In terms of implications on donor site functionality and esthetics, the need for further research within this area is therefore justifiable [41].

Volumetric reductions of flaps are prevalent up to three years post reconstruction [44], affecting esthetic and functional outcomes. Although free flap reconstruction in head and neck cancer surgery follows a standardized surgical approach, there is no consensus procedure on estimation of flap volumes [45].

Since there are no previous studies on the comparison of volumes between preoperative tumor of the tongue and postoperative RFFF, no direct comparisons to other research can be drawn. However, past literature on both the topic of volumetric measurements using ITK-SNAP as well as RFFF coverage after oral tongue cancer will be discussed in the following section to provide an overview of the research currently available within this field.

Bozec et al. found that reconstruction with RFFF and radical ablative surgery provide promising oncologic and functional results in patients with oral cancer. Three out of 132 patients within this study suffered from RFFF failures. [33].

A more recent study by Xu et al. compares data from five studies comparing donor-site outcomes of RFFF and UFFF. This analysis showed a significantly lower incidence of complications at the donor site of UFFF than that in RFFF. They suggest the UFFF may be an ideal substitute for RFFF in reconstructive surgery; however, large-scale studies with long-term follow up are necessary to confirm these findings [46].

Regarding volumetric quantifications with ITK-SNAP, several authors have successfully conducted research on various structures using semi-automatic segmentation.

Zirk et al. conducted a retrospective study, analyzing differences in volumes of areas of osteolysis caused by medication-related osteonecrosis of the jaw (MRONJ). They found that the most frequently affected localization is the mandible, with female subjects showing significantly more lesions within the maxilla. Their volumetric measurements using ITK-SNAP revealed a greater absolute osteolysis volume in male subjects [47].

Another study by Ritschl et al. looked at bone volume behavior of free fibula and iliac crest flaps using ITK-SNAP. 113 cases in total were analyzed and showed a stability, whilst iliac crest flaps showed a higher bone volume reduction. Several factors contributed significantly to bone volume behavior including the time interval between operation and CT scan, age, gender, reconstruction with the flap, and the number of fibula segments [34].

A more recent study by Younes et al. compared flap shrinkage between the RFFF and the ALTF (anterolateral thigh free flap). Whilst flap shrinkage was shown for both flaps, the RFFF showed a significantly higher percentage shrinkage than the ALTF. The authors suggest that in hemiglossectomy cases, the ALTF is made 1.4 times larger than the defect, while the RFFF is made 1.5 times larger than the defect [48].

Doubtlessly a larger tumor volume resection will lead to a larger flap volume, thus leaving a larger defect at the donor site. Limitations of volumetric predictions using 3-Dimensional images include an increased distortion rate of soft tissue and discrepancies between estimated resection margins using radiographic information and actual resection margins intraoperatively. Since resection for R0 needs to be performed in cancer-free tissue, these volumes tend to be larger than initially anticipated. Further volume changing effects include surgical complications such as edema or the priorization of functional and anatomical characteristics. These include rebuilding anatomical conjunctions such as the floor of the mouth, or reconstruction of tongue movement by prioritizing the repair of the length of the tongue. Overcoming these limitations may lead to more accurate definitions of tumor margins preoperatively and could aid in predicting the anticipated flap volume more precisely. This would allow for smaller extraction volumes from the donor site and lead to decreased donor site morbidity, as well as higher patient satisfaction.

## Conclusion

In summary, the extracted data shows a significant positive correlation between preoperative tumor volume and postoperative flap volume (*p* = 0,769 (*p* < 0.001)). Nonetheless, implications within this study include the absence of other studies as direct comparisons, as well as the lack of bigger amounts of data leading to a small sample size. Due to the small sample size within this study, a reproducible numeric constant can’t be directly deducted to describe a relationship between pre-operative tumor volume and required flap size. A larger sample size may increase the reliability of statistical relationships and allow more accurate estimations on flap volume based on primary tumor volume. A positive correlation between tumor Volume and flap volume highlights the importance of efficient flap planning with increasing tumor volume. A larger extraction volume of the radial forearm free flap from the donor site, compromises the forearm more and results in higher rates of donor site morbidity. This implication highlights the clinical relevance of this investigation and justifies the cause for further clinical studies to be carried out within this area of research.

## Data Availability

No datasets were generated or analysed during the current study.
